# Diagnostic comparison between FECPAK^G2^ and the Kato-Katz method for analyzing soil-transmitted helminth eggs in stool

**DOI:** 10.1371/journal.pntd.0006562

**Published:** 2018-06-04

**Authors:** Wendelin Moser, Oliver Bärenbold, Greg J. Mirams, Piet Cools, Johnny Vlaminck, Said M. Ali, Shaali M. Ame, Jan Hattendorf, Penelope Vounatsou, Bruno Levecke, Jennifer Keiser

**Affiliations:** 1 Department of Medical Parasitology and Infection Biology, Swiss Tropical and Public Health Institute, Basel Switzerland; 2 University of Basel, Basel, Switzerland; 3 Department of Epidemiology and Public Health, Swiss Tropical and Public Health Institute, Basel, Switzerland; 4 Techion Group Limited, Dunedin, New Zealand; 5 Department of Virology, Parasitology and Immunology, Faculty of Veterinary Medicine, Ghent University, Ghent, Belgium; 6 Laboratory Division, Public Health Laboratory-Ivo de Carneri, Chake Chake, Tanzania; Univeristy of Torino, ITALY

## Abstract

**Background:**

Over one billion people are infected with soil-transmitted helminths (STH), i.e. *Ascaris lumbricoides*, hookworm and *Trichuris trichiura*. For estimating drug efficacy and monitoring anthelminthic drug resistance, accurate diagnostic methods are critical. FECPAK^G2^ is a new remote-diagnostic tool used in veterinary medicine, which produces an image of the stool sample that can be stored on an internet cloud. We compared for the first time FECPAK^G2^ with the recommended Kato-Katz method.

**Methodology/Principal findings:**

Two stool samples were collected from adolescent participants (age 15–18 years) at baseline and 14 to 21 days after treatment in the framework of a randomized clinical trial on Pemba Island, Tanzania. Stool samples were analyzed with different diagnostic efforts: i) one or ii) two Kato-Katz thick smears from the first sample, iii) two Kato-Katz thick smears from two samples and iv) FECPAK^G2^ from the first sample. Parameters were calculated based on a hierarchical Bayesian egg count model.

Complete data for all diagnostic efforts were available from 615 participants at baseline and 231 hookworm-positive participants at follow-up. At baseline FECPAK^G2^ revealed a sensitivity of 75.6% (72.0–77.7) for detecting *A*. *lumbricoides*, 71.5% (67.4–95.3) for hookworm and 65.8% (64.9–66.2) for *T*. *trichiura*, which was significantly lower (all p<0.05) than any of the Kato-Katz methods and highly dependent on infection intensity. Despite that the egg counts based on FECPAK^G2^ were relatively lower compared to Kato-Katz by a ratio of 0.38 (0.32–0.43) for *A*. *lumbricoides*, 0.36 (0.33–0.40) for hookworm and 0.08 (0.07–0.09) for *T*. *trichiura*, the egg reduction rates (ERR) were correctly estimated with FECPAK^G2^.

**Conclusions/Significance:**

The sensitivity to identify any STH infection was considerably lower for FECPAK^G2^ compared to Kato-Katz. Following rigorous development, FECPAK^G2^ might be an interesting tool with unique features for epidemiological and clinical studies.

## Author summary

About 1.5 billion people are infected with soil-transmitted helminths (*Ascaris lumbricoides*, hookworm and *Trichuris trichiura*). Since morbidity correlates with the number of worms harbored by an infected individual, WHO aims to reduce moderate and heavy infections in pre- and school-aged children by 2020. The cornerstone of estimating the prevalence, assessing drug efficacy and monitoring drug resistance are accurate diagnostic tools. The currently recommended Kato-Katz, has some major disadvantages like a short processing window and low sensitivity and new diagnostic tools are needed. FECPAK^G2^ is an online, remote location tool developed for counting nematode eggs in sheep, cattle, equine and Camelids fecal samples. The output of the system is an image of the sample, which is saved and uploaded onto an internet cloud. This offers new options particularly for low resource settings. We tested FECPAK^G2^ for the first time for analyzing human stool in a randomized controlled trial. We observed a baseline sensitivity of 75.6% for detecting *A*. *lumbricoides*, 71.5% for hookworm and 65.8% for *T*. *trichiura* and an increased sensitivity for moderate infection intensities. Despite lower sensitivity and egg counts, FECPAK^G2^ was able to correctly estimate egg reduction rates. Following further development, FECPAK^G2^ might become an important tool for soil-transmitted helminth control programs, epidemiological and clinical studies.

## Introduction

Approximately 1.5 billion people are infected with the soil-transmitted helminths (STH) *Ascaris lumbricoides*, hookworm and/or *Trichuris trichiura* [1]. While the majority of light infections remain asymptomatic, moderate and heavy infections are responsible for a considerable health burden, including growth stunting, intellectual retardation, cognitive and educational deficits, malnutrition and iron-deficiency anemia [2,3]. The estimated global STH burden was 3.3 million disability adjusted life-years in 2016 [4]. Large scale distribution of anthelminthic drugs (i.e. albendazole and mebendazole) to at-risk populations in preventive chemotherapy programs is the current strategy against STH infections [5]. The ultimate goal of the World Health Organization (WHO) is to reduce burden caused by moderate and heavy infections [5].

For estimating prevalence of soil-transmitted helminthiasis, assessing infection intensities, evaluating drug efficacy and monitoring drug resistance, accurate diagnostic methods are essential [5–7]. The currently recommended Kato-Katz method has already been in use for decades [8,9]. The advantages of Kato-Katz are its low cost, short sample preparation time, simple handling and the need of only basic equipment [8,10]. However, the method has a low sensitivity for low STH infection intensities, hookworm eggs disappear after one hour and samples and slides for hookworm cannot be stored [11–13]. The sensitivity can be improved by analyzing multiple Kato-Katz thick smears from several samples [12,14] or by analyzing an increased amount of stool as it is done by the FLOTAC (1 gram) or Mini-FLOTAC (2/10 gram) system [15,16].

Once the strategy is moving towards transmission control and STH elimination, an increased sensitivity of the diagnostic method of choice is crucial [6]. Nowadays, several molecular tools are available to diagnose STH infections. Although these tools show increased sensitivity, they are time consuming, require costly laboratory equipment and highly skilled laboratory technicians [17,18]. Therefore, the research on new diagnostic tools is necessary, with the aim of developing a fast, simple and cost-effective method for the diagnosis of STH infections. FECPAK^G2^ is an online, remote location, parasite diagnostic system used in veterinary medicine [19]. The first FECPAK system was originally established for counting nematode eggs in sheep fecal samples [20–22]. FECPAK^G2^ is based on the flotation-dilution principle, similar to the McMaster method [23]. The novelty of FECPAK^G2^ is the accumulation of parasite eggs into one viewing area within a fluid meniscus [24,25]. An image of the fecal sample is then captured, is stored offline on a computer and can be uploaded onto a cloud once connected to the internet. Subsequently, the image can be analyzed at any time by specialists around the world.

The aim of the study was to comparatively assess the sensitivity, the associated cure rates (CRs), the egg counts and their related egg reduction rates (ERR) based on FECPAK^G2^ and the Kato-Katz method (i.e. single, double and quadruplicate Kato-Katz). The diagnostic comparison was conducted in the framework of a clinical trial including different tribendimidine co-administrations against hookworm infections on Pemba Island, Tanzania [26].

## Methods

### Ethics statement

In 2016, a randomized controlled, single-blind, non-inferiority trial evaluating the efficacy of tribendimidine co-administrations, was conducted in Tanzania and Côte d’Ivoire. The presented data on the diagnostic comparison is based exclusively on samples collected in Tanzania [26]. Ethical clearance was obtained from the Zanzibar Medical Research and Ethical Committee in Tanzania (reference ZAMREC/0001/APRIL/016) and the Ethics Committee of Northwestern and Central Switzerland (reference EKNZ UBE-15/35). This trial is registered with ISRCTN registry (number ISRCTN14373201). Written informed consent from parents or legal guardians and verbal assent from participants were obtained prior to the sample collection. At the end of the study, participants remaining positive for any STH were treated with a standard dose albendazole (400 mg) according to national guidelines [27].

### Study population

The study was carried out during August and September 2016 on Pemba Island, Tanzania. Details of the clinical trial procedure are described elsewhere [26]. Briefly, adolescents (age 15 to 18) from four different secondary schools (Wingwi, Mizingani, Wesha and Tumbe) were asked to provide two stool samples at baseline. Hookworm positive participants were randomly allocated to the treatment arms: i) tribendimidine (400 mg), ii) tribendimidine (400 mg) plus ivermectin (200 μg/kg), iii) tribendimidine (400 mg) plus oxantel pamoate (25 mg/kg) and iv) albendazole (400 mg) plus oxantel pamoate (25 mg/kg). Another two stool samples were collected 14 to 21 days after treatment at the follow-up visit. Participants, laboratory and field technicians were blinded.

### Parasitological methods

#### Kato-Katz

Fresh stool samples were labelled with a unique identification number and transferred to the Public Health Laboratory-Ivo de Carneri. Of each stool sample, a duplicate Kato-Katz thick smear using a 41.7 mg template [9], was prepared by experienced laboratory technicians. Between a half and one hour after preparation–to avoid over-clearing of hookworm eggs [13]–the STH eggs were counted using a light microscope. For assuring diagnostic quality, 10% of all Kato-Katz slides were randomly selected, re-examined by the study investigator for *A*. *lumbricoides* and *T*. *trichiura* eggs. In case of discordant results the slides were read a third time and discussed until consensus was reached [28].

#### FECPAK^G2^

The first stool sample collected at baseline and follow-up was analyzed with FECPAK^G2^. The standard operational procedure (SOP) manual was adopted for human stool samples by Ayana and colleagues and is made available online [25]. Briefly, three grams from each stool sample were mixed thoroughly with 38 ml tap water using a Fill-FLOTAC [16]. The suspension was transferred to the FECPAK^G2^ sedimenter and tap water was added. After one hour the supernatant was flushed away and 80 ml saturated NaCl flotation solution (density = 1.2) was added to the sediment, giving a total volume of 95 ml, which equates to 0.032 g stool per ml saline. The solution was transferred to the FECPAK^G2^ cylinder, which includes two wire mesh sieves (apertures: outer 425 microns, inner 250 microns) to remove large debris. The two wells of the FECPAK^G2^ cassette were each filled with each 455 μl of the solution which combined contained 0.029 g stool. After 20 minutes, the cassette was placed into the MICRO-I (FECPAK^G2^ imaging unit) and a single image frame of the axisymmetric meniscus of each well was captured [29]. The images were uploaded onto the Microsoft Azure Cloud system (Microsoft Corp., Redmond, WA) via the FECPAK^G2^ software. The mark-up of the images was done by two laboratory technicians on Pemba, using the FECPAK^G2^ software. STH eggs were identified on both images, marked according to the species and the combined total egg count was automatically determined by the FECPAK^G2^ software. Quality control was performed on half of the images in Switzerland using a computer-generated list. An image was classified as insufficient quality and excluded in case of: blurriness, stacking bands, cracked rods, debris, air bubbles, over and under filling of the cassette wells.

### Statistical analysis

For each of the following diagnostic method i) one Kato-Katz thick smear of the first sample, ii) two Kato-Katz thick smears of the first sample, iii) quadruplicate Kato-Katz thick smears (two Kato-Katz thick smears of each sample) and iv) FECPAK^G2^ from the first sample, the sensitivity was determined for *A*. *lumbricoides*, hookworm and *T*. *trichiura* at baseline and follow-up. The sample size calculated for the clinical trial [26] was deemed sufficient for this diagnostic comparison.

A hierarchical Bayesian egg-count model as described by Bärenbold et al. [30] was applied to individual level data. The Kato-Katz counts were modelled with a negative binomial distribution depending on the daily egg density. The log of the mean egg density at the individual level was assumed to vary normally between days and the mean infection intensities to be gamma distributed in the population with a mean that reflects the mean infection intensity of an infected individual. The model was extended with a negative binomial process, to simulate the data obtained by FECPAK^G2^, with a linearly reduced daily egg density for the same individual compared to Kato-Katz and an independent over-dispersion parameter of the negative binomial distribution. Sample sensitivity of each test was calculated as the ratio between observed prevalence and estimated true prevalence. We assumed a specificity of more than 98% for Kato-Katz and set an uniform prior for the specificity of FECPAK^G2^.

The efficacy for each treatment arm in terms of CRs (percentage of egg-negative participants with a previous infection) and ERRs (percentage of arithmetic mean egg count reduction from baseline to follow-up) was calculated according to the four different diagnostic methods for all baseline positive children. CRs were calculated with imperfect diagnostic methods and an estimate for the true value based on the egg count model was given. Varying sensitivity between baseline and follow-up because of reduced infection intensity, show the following relation to the “true” CRs: $(1-{CR}_{True})=(1-{CR}_{observed})\times \frac{s_{bl}}{s_{fu}}$ which follows from the definition of the cure rate under the assumption of no reinfections happening between baseline and follow-up (S1 Text). For the different diagnostic methods, the sensitivity-ratio between baseline and follow-up was calculated. In case the 95% confidence interval (CI) of the sensitivity-ratio included 1, the apparent CRs were not significantly different from the true CR.

Eggs per gram of stool (EPG) were calculated by multiplying the single and the average of two (duplicate) or four (quadruplicate) Kato-Katz thick smears with a factor of 24. For FECPAK^G2^ the egg counts were multiplied by a factor of 34. The true ERR was based on the reduction from baseline to follow-up of the mean infection intensity estimates from the model. The 95%-confidence intervals (CI) for the apparent ERRs of the treatments for each diagnostic method were obtained using a bootstrap resampling approach with 5000 replications [31].

For the statistical analysis, Stata version 14.0 (Stata Corporation; College Station; Texas, United States of America), OpenBugs version 3.2.3, Stan version 2.16.2, and R version 3.4.1 were used.

## Results

### Study flow

**Fig 1:**
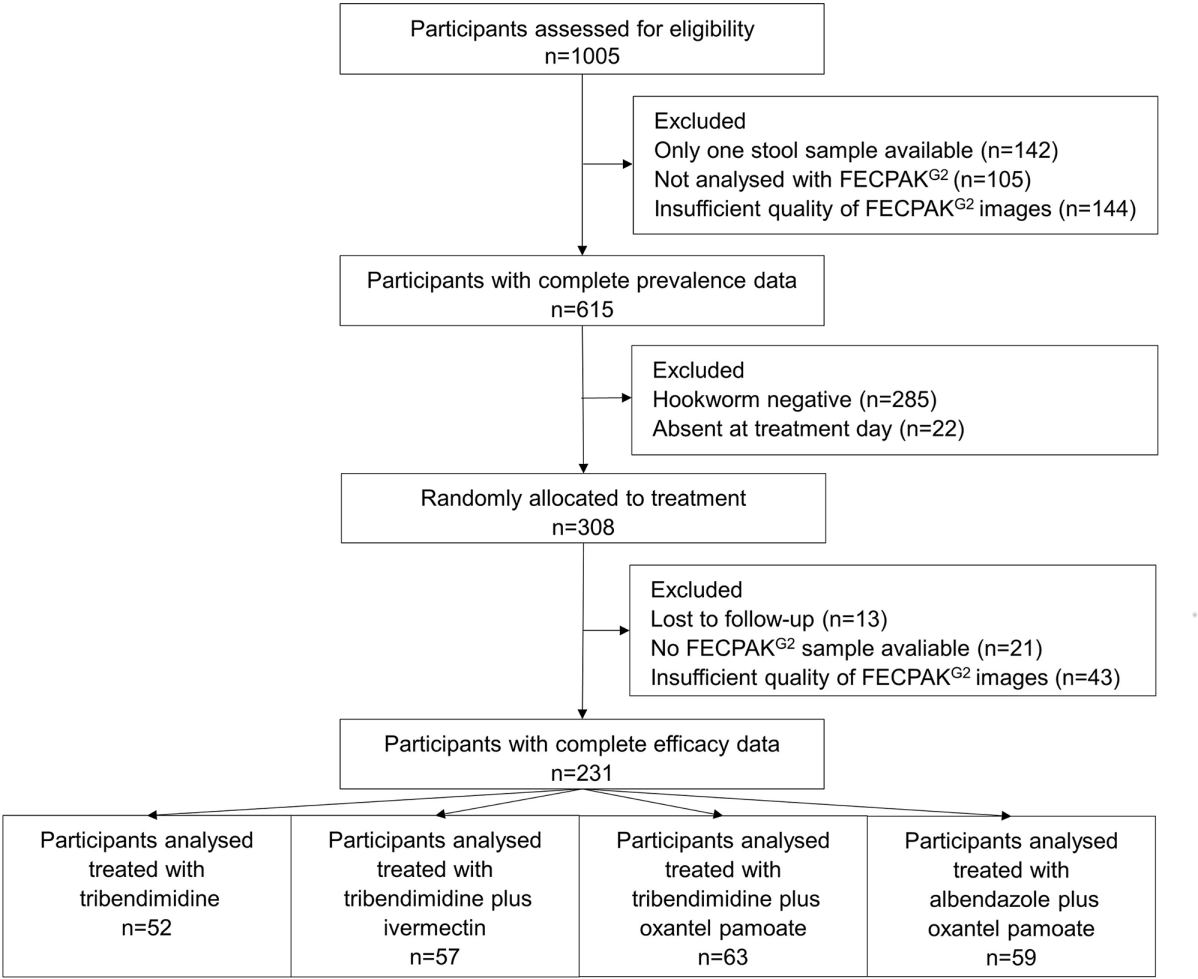
Study flow of stool samples collection and analysis using the single, duplicate, quadruplicate Kato-Katz and FECPAK^G2^.

Stool samples from 1,005 participants were collected (Fig 1). Data of 391 participants were excluded: 142 provided only one stool sample, the sample of 105 participants were not analyzed with FECPAK^G2^ because of technical issues (ID mismatch or not sufficient stool) and FECPAK^G2^ images from 144 from participants were classified as insufficient quality. A total of 615 participants had complete baseline data and 384, 330 and 579 were infected with *A*. *lumbricoides*, hookworm and *T*. *trichiura*, respectively (Table 1). Only 25 participants were negative for any STH. From the participants with baseline data, 308 were treated, whereas 285 were hookworm negative and 22 were absent at treatment day. Of 308 participants randomized to treatment 13 participants were lost to follow-up,. from 21 participants the samples were not analyzed with FECPAK^G2^ because of technical issues and the data of 43 participants were excluded because of insufficient quality of the images. Complete follow-up data were available from 231 participants.

**Table 1:** Estimated true prevalence, sensitivity and arithmetic mean egg counts from the 615 participants with complete baseline data according to the four different diagnostic methods.

		*A*. *lumbricoides*	Hookworm	*T*. *trichiura*
Estimated true	Prevalence	64.0 (62.2–67.1)	54.8 (53.1–57.9)	94.7 (94.0–96.0)
	Eggs per gram of stool	18125 (15024–21724)	474 (402–558)	1999 (1762–2252)
Single Kato-Katz	No. of positive participants (%)	347 (56.4)	288 (46.8)	553 (89.9)
	Eggs per gram of stool	14361 (12099–16622)	509 (415–603)	1760 (1517–2003)
	Sensitivity	87.8 (83.6–90.7)	85.5 (80.4–88.1)	94.8 (93.3–95.6)
Duplicate Kato-Katz	No. of positive participants %	353 (57.4)	299 (48.6)	559 (90.9)
	Eggs per gram of stool	14175 (11866–16485)	474 (391–556)	1725 (1489–1961)
	Sensitivity	89.8 (85.6–92.3)	89.1 (84.0–91.6)	96.1 (94.7–96.7)
Quadruplicate Kato-Katz	No. of positive participants (%)	384 (62.4)	330 (53.7)	579 (94.2)
	Eggs per gram of stool	13478 (11435–15521)	434 (359–508)	1796 (1544–2048)
	Sensitivity	97.7 (93.1–99.9)	98.3 (92.7–99.9)	99.5 (98.1–99.9)
FECPACK^G2^	No. of positive participants (%)	297 (48.3)	240 (39.0)	383 (62.3)
	Eggs per gram of stool	3048 (2501–3595)	245 (197–293)	171 (148–194)
	Sensitivity	75.6 (72.0–77.7)	71.5 (67.4–95.3)	65.8 (64.9–66.2)
	Specificity	96.9 (94.8–98.9)	91.3 (89.3–93.1)	95.3 (91.8–97.6)

Numbers in brackets show 95% confidence interval, unless otherwise indicated

### True prevalence, sensitivity and specificity

The estimated true baseline prevalence was 64.0% (95% confidence interval [CI] 62.2–67.1) for *A*. *lumbricoides*, 54.8% (53.1–57.9) for hookworm and 94.7% (94.0–96.0) for *T*. *trichiura*. At follow-up, prevalence values of 5.5% (4.0–8.5), 44.3% (39.4–50.5) and 52.0% (49.8–54.7) were estimated for *A*. *lumbricoides*, hookworm and *T*. *trichiura* respectively (S1 Table).

At baseline, the sensitivity of the quadruplicate Kato-Katz was significantly higher compared to any other method with 97.7% (93.1–99.9) for *A*. *lumbricoides*, 98.3% (92.7–99.9) for hookworm and 99.5% (98.1–99.9) for *T*. *trichiura*. In contrast, the sensitivity of FECPAK^G2^ was significantly lower than the single and duplicate Kato-Katz method (all p<0.05) with 75.6% (72.0–77.7) for detecting *A*. *lumbricoides*, 71.5% (67.4–95.3) for hookworm and 65.8% (64.9–66.2) for *T*. *trichiura*. The specificity estimated for FECPAK^G2^ was 96.9% (94.8–98.9) for *A*. *lumbricoides*, 91.3% (89.3–93.1) for hookworm and 95.3% (91.8–97.6) for *T*. *trichiura*. Estimated true prevalence, sensitivities, sensitivity-ratio and egg counts from the 231 participants with complete follow-up data is presented in S1 Table.

**Fig 2:**
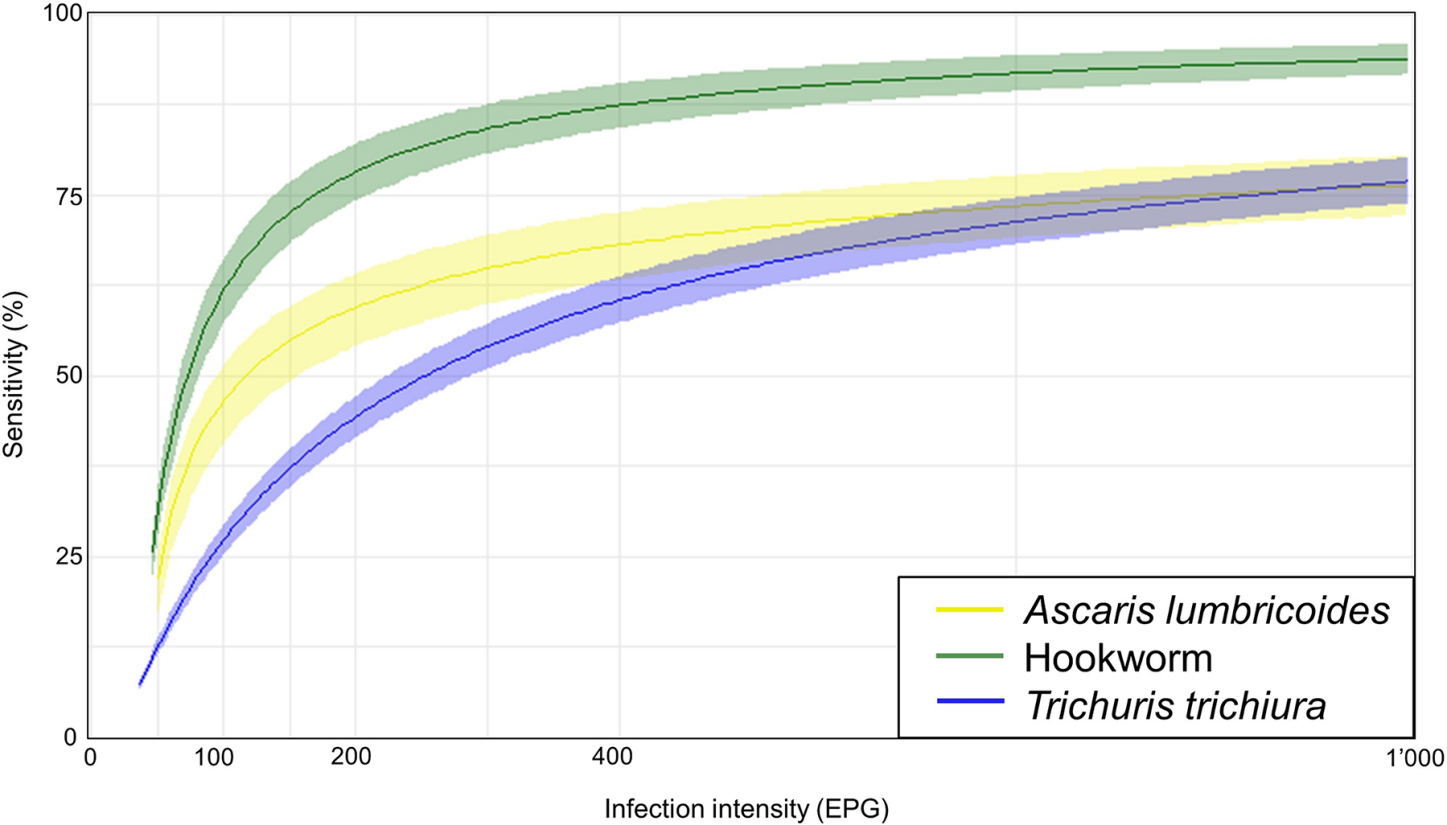
The estimated sensitivity of FECPAK^G2^ based on the infection intensity.

The sensitivity of FECPAK^G2^ was highly dependent on the infection intensity (Fig 2, S2 Table S). For an infection intensity of 100 EPG, the sensitivity of FECPAK^G2^ was as low as 42.9% (37.3–46.9) for *A*. *lumbricoides*, 56.3% (51.0–61.3) for hookworm and 22.2% (19.9–23.5) for *T*. *trichiura*. The estimated sensitivity increased for moderate infection intensity according to WHO cut-offs [8] and resulted in 82.0% (78.8–84.5) for *A*. *lumbricoides* (EPG 5000), 95.6% (94.1–97.3) for hookworm (EPG 2000) and 70.3% (67.6–73.9) for *T*. *trichiura* (EPG 1000).

### Estimation of egg counts

**Fig 3:**
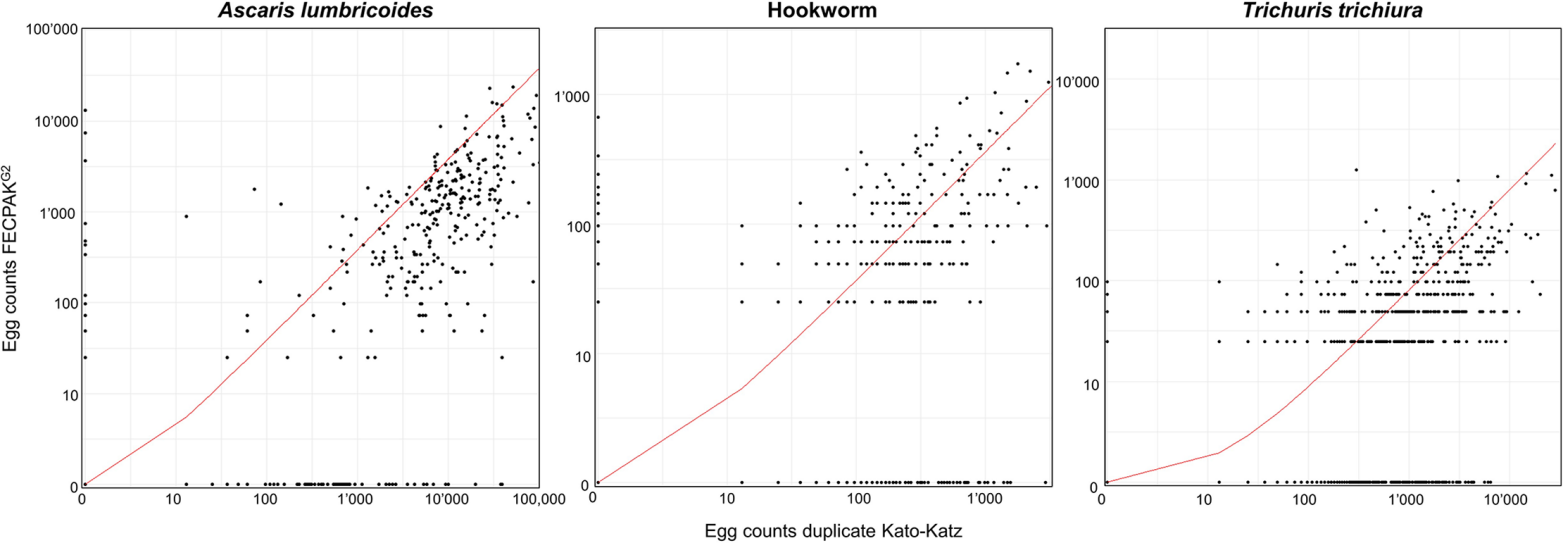
Scatter plot of the egg counts based on FECPAK^G2^ and duplicate Kato-Katz egg counts. Red line indicates egg density-ratio between Kato-Katz and FECPAK^G2^.

The estimated true mean egg counts according to the model were 18125 EPG (15024–21724) for *A*. *lumbricoides*, 474 EPG (402–558) for hookworm and 1999 EPG (1762–2252) for *T*. *trichiura* at baseline (Table 1). Data from the follow up is presented in S1 Table. The EPGs based on FECPAK^G2^ were several times lower at baseline and follow-up compared to the different Kato-Katz sampling efforts. Relative to the Kato-Katz, the egg counts of FECPAK^G2^ were lower by an egg density-ratio (Fig 3, red line) of 0.38 (0.32–0.43) for *A*. *lumbricoides*, 0.36 (0.33–0.40) for hookworm and 0.08 (0.07–0.09) for *T*. *trichiura*.

### Cure rates

**Fig 4:**
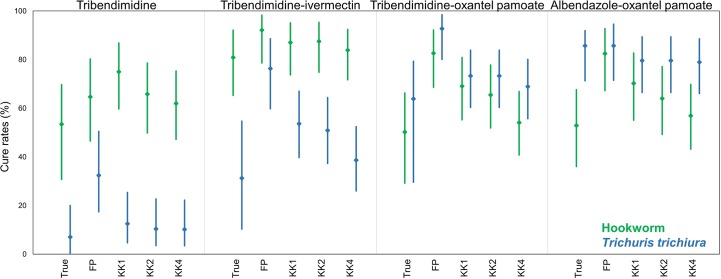
True cure rates (True) and cure rates based on a single (KK1) duplicate (KK2), quadruplicate Kato-Katz (KK4) and FECPAK^G2^ (FP) against hookworm and *T*. *trichiura* for the four different treatment arms. Cure rates against *A*. *lumbricoides* are not presented.

The true CRs estimated by the model and the apparent CRs according to the different diagnostic methods are presented in Fig 4 and S3 Table. According to the sensitivity-ratio (SR), there was no noteworthy difference between the true estimated and the apparent CRs for the quadruplicate Kato-Katz (S3 Table). For FECPAK^G2^ the true estimated CRs for hookworm (SR 2.21, 1.88–2.63) and *T*. *trichiura* (SR 2.06, 1.83–2.36) differed significantly compared to the true estimated CRs. Since the CRs were generally high for *A*. *lumbricoides* (CR>93%) and most participants were cured, the sensitivity-ratio estimates had a higher uncertainty, included one and no differences among the diagnostic method were observed (SR 1.38, 0.98–2.28, S3 Table).

For tribendimidine or albendazole in combination with oxantel pamoate against hookworm, low true CRs were observed and the apparent CRs decreased with higher Kato-Katz sampling effort. The CRs according to FECPAK^G2^ compared to the true CRs were significantly higher for tribendimidine-oxantel pamoate (82.6%, 68.6–92.2 *versus* 46.3%, 35.2–52.6) and albendazole-oxantel pamoate (82.5%, 67.2–92.7 *versus* 49.2%, 36.7–56.2). Against *T*. *trichiura*, the difference was particularly pronounced for the treatment arm tribendimidine-ivermectin with a true CRs of 34.1% (25.7–37.7), followed by the quadruplicate (38.6%, 26.0–52.4) and duplicate Kato-Katz (50.9%, 37.3–64.4) and a significantly higher CR for FECPAK^G2^ (76.3%, 59.8–88.6). Similar, slightly less pronounced differences were found between the true and the FECPAK^G2^ CRs for tribendimidine monotherapy (5.5%, 1.6–8.5 *versus* 32.4%, 17.4–50.5) and tribendimidine-oxantel pamoate (66.8%, 58.1–71.1 *versus* 92.7%, 80.1–98.5).

### Egg reduction rate according to diagnostic methods

**Fig 5:**
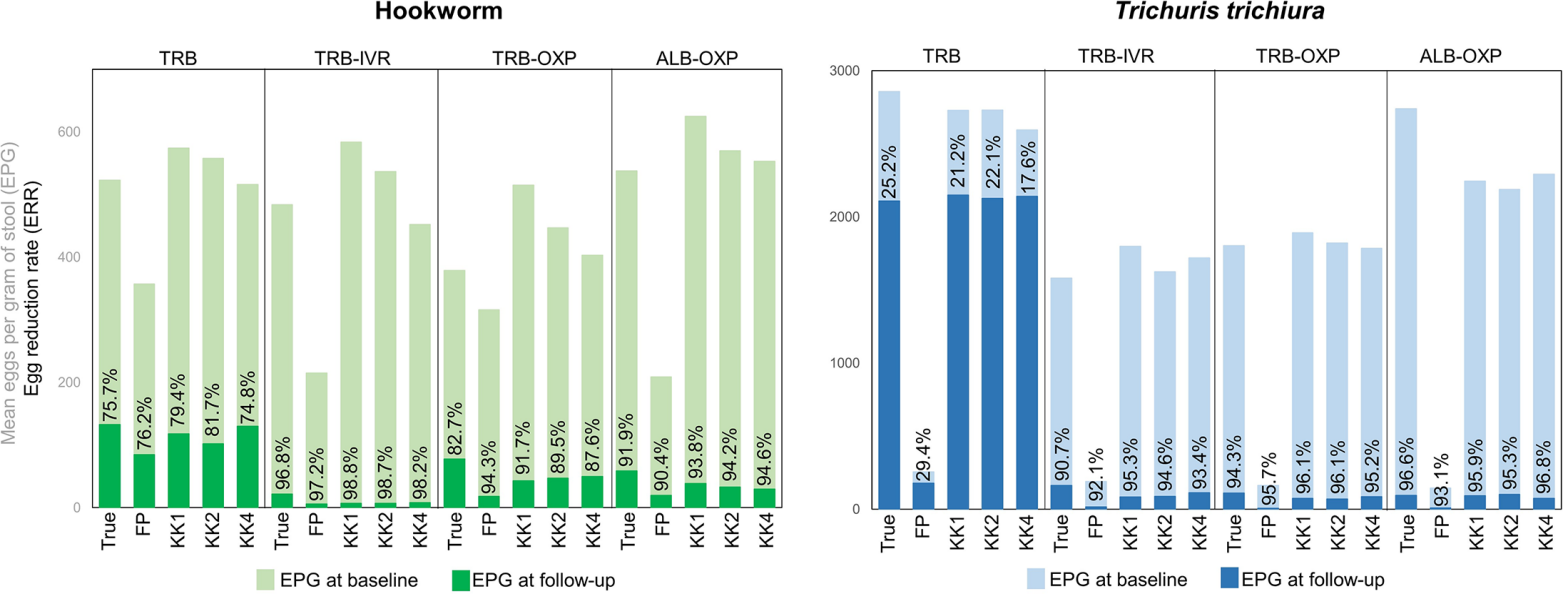
True egg reduction rates (ERR) and ERRs based on single (KK1), duplicate (KK2), quadruplicate Kato-Katz (KK4) and FECPAK^G2^ after treatment with tribendimidine (TRB), tribendimidine-ivermectin (TRB-IVR), tribendimidine-oxantel pamoate (TRB-OXP) and albendazole-oxantel pamoate (ALB-OXP). Egg reduction rates against *A*. *lumbricoides* are not presented.

No noteworthy difference was observed between the true ERRs and the arithmetic ERRs according to the four diagnostic methods (S4 Table, Fig 5). Despite lower EPGs for FECPAK^G2^ compared to any of the Kato-Katz methods, the ERRs and interval estimates remained similar with one exception. For tribendimidine monotherapy against *T*. *trichiura*, the true ERR (22.9%, 5.3–50.3) and the ERR determined by FECPAK^G2^ (29.4%, -38.3–66.7), were non-significantly higher compared to the ERRs based on the quadruplicate Kato-Katz (17.6%, -17.1–38.8).

## Discussion

New diagnostic tools are required to complement or replace the currently recommended Kato-Katz method [8]. FECPAK^G2^ is a remote-location, online parasite diagnostic system, which is used in veterinary medicine. This is the first study, which compared the FECPAK^G2^ method in human parasitology in the framework of a randomized, clinical trial on Pemba island, Tanzania [26]. We assessed for FECPAK^G2^ several different diagnostic parameters including prevalence, sensitivity and the associated CRs, egg counts, infection intensity and the related reduction in intensity after treatment.

For FECPAK^G2^, sensitivity was significantly lower compared to single, duplicate and quadruplicate Kato-Katz for identifying any of the STH at baseline and follow-up. However, a lower sensitivity was expected, since FECPAK^G2^ examines only 1/34 of gram of stool compared to 1/24 gram for the single, 1/12 gram for duplicate and 1/12 (day 1) plus 1/12 gram (day 2) for the quadruplicate Kato-Katz. For detecting moderate infection intensities, the FECPAK^G2^ sensitivity increased to 82.0% for *A*. *lumbricoides*, 95.6% for hookworm and 70.3% for *T*. *trichiura*. Similar characteristics have been shown for the Kato-Katz method, i.e. low sensitivity for low infection intensities and high sensitivity for moderate and heavy infections [12].

Since the CRs are a function of the sensitivity, and the sensitivity of FECPAK^G2^ was highly dependent on the infection intensity, the FECPAK^G2^ CRs and the true CRs were significantly different. For example, for tribendimidine-oxantel pamoate the *T*. *trichiura* infection intensity changed from baseline (true EPG~2000) to follow-up (true EPG~100), which led to a decreased sensitivity from 80.5% (baseline) to 22.2% (follow-up,). Therefore, the CR for FECPAK^G2^ (92.7%) was significantly overestimated compared to the true CR (66.8%) (S3 Table). These results indicate, that in the present form FECPAK^G2^ does not accurately estimate CRs, which was also true for the single and duplicate Kato-Katz.

While the lower sensitivity negatively influenced the CRs, the ERRs remained unchanged, which was already reported by Levecke and colleagues for different Kato-Katz sampling efforts [32]. Similarly, no differences among the diagnostic methods were shown in our study. For instance, the above-mentioned treatment example resulted in a true ERR of 94.3%, which was not significantly different from an ERR of 95.7% with FECPAK^G2^(S4 Table). While the egg counts with FECPAK^G2^ were generally lower compared to Kato-Katz, the ERRs remained equal. Thus, FECPAK^G2^ might be an interesting tool for monitoring anthelmintic drug efficacy [5].

A lower egg recovery rate from sheep or cattle fecal samples was already observed for the earlier FECPAK system in comparison with FLOTAC, Mini-FLOTAC and McMaster, however, no data about the performance of the new FECPAK^G2^ was available [20,21]. The lower recovery of eggs by FECPAK^G2^ might be due to the inability of detecting unfertilized *A*. *lumbricoides* eggs and a high extent of debris covering the eggs. To overcome the problem with high debris, a variety of different sized meshes for the FECPAK^G2^ cylinder are currently being tested. In addition, in the FECPAK^G2^ cassette the capillary rise of the aqueous saline generates an axisymmetric meniscus over the cylindrical rod, which converges the eggs on the top of the meniscus [29]. The accumulated eggs remain in a single microscopic field of view and a staged image of the meniscus is taken with the MICRO-I. For increasing the recovery, a vibration function in the MICRO-I might improve the egg accumulation, as suggested by Sowerby and colleagues [29]. Further optical and image processing improvements for the MICRO-I are under development. These improvements will speed up the processing capability of the device and will generate higher quality images that are expected to improve the egg recovery (sensitivity) and accuracy of the image mark-up.

Obviously, the examination of only one cassette and one stool sample with FECPAK^G2^ was a limitation of our study. The collection of two stool samples would account for the day-to-day variation and would increase sensitivity [30]. For example, in this study the sensitivity increased from one analyzed stool sample (single or duplicate Kato-Katz) to two stool samples (quadruplicate Kato-Katz) about 10%-points for *A*. *lumbricoides* and hookworm. The sensitivity-ratio indicated a weak dependence of the quadruplicate Kato-Katz on infection intensities, which did not induce a significant bias for this study, since the sample size was rather small and precision estimates wide. Nevertheless, the bias might become important in larger studies with higher accuracy. By collecting samples on several days, the sensitivity of FECPAK^G2^ for low infection intensities might improve, which would limit the bias introduced in CR estimates. Hence, the analysis of two cassettes and two stool samples with FECPAK^G2^, should be the subject of further studies. Additionally, the time for preparing one sample and the costs of FECPAK^G2^ should be compared against current established diagnostic methods.

Other limitations of this study were the loss of samples due to the mixing up of sample IDs, insufficient amount of stool and insufficient quality of many FECPAK^G2^ images. In more detail, a total of 144 (19.0%) samples at baseline and 43 (14.0%) samples at follow-up were excluded, because of insufficient filling of the cassette or problems associated with the capturing of the image (i.e. blurriness, stacking bands, cracked rods, debris, air bubbles etc.), which was detected only during the mark-up process of the images when sample analysis could not be repeated. With lower numbers of analyzed samples per day, larger number of laboratory technicians, better experience with handling of the FECPAK^G2^ the number of excluded samples might have been lower and hence these factors should be considered in future studies.

Despite the discussed limitations of FECPAK^G2^ at the current stage of development, several advantages are worth highlighting. The most innovative feature is the captured image, which is saved offline, uploaded online onto an internet cloud and analyzed at any later time point. In contrast, the major limitation of Kato-Katz is the disappearance of hookworm eggs one hour after the preparation [13]. Moreover, stool samples cannot be stored [11], which limits the time to control the diagnostic quality [28]. The storage of the FECPAK^G2^ images offers new options, especially for low resource settings. First, diagnostic results of STH can be stored for the first time, analyzed by trained technicians around the world and quality control is not restricted to time. Second, technicians can focus on processing the samples while analysis is done at a later time point, potentially leading to a faster turnaround in laboratories. Third, in case of identification discrepancies, specialist around the world can be consulted, which improves the diagnostic results. Research is ongoing to develop an image-analysis algorithm, which will automatically count the different helminth eggs in the future.

In conclusion, we have assessed for the first time the performance of FECPAK^G2^ in human parasitology, in the framework of a randomized controlled trial. Compared to different Kato-Katz sampling efforts, FECPAK^G2^ showed lower sensitivities and egg recovery rates. The sensitivity increased with higher infection intensities. Further research is required for increasing sensitivity and egg recovery to develop FECPAK^G2^ as a useful addition in the near future to the depleted diagnostic set of tools for STH infections.

## Supporting information

S1 Checklist (DOCX)

S1 Text (DOCX)

S1 Table (DOCX)

S2 Table (DOCX)

S3 Table (DOCX)

S4 Table (DOCX)
