# Supplementary material for: Diagnostic comparison between FECPAKG2 and the Kato-Katz method for analyzing soil-transmitted helminth eggs in stool
Source: PLoS Negl Trop Dis. 2018 Jun 4;12(6):e0006562. doi: 10.1371/journal.pntd.0006562 (PMC6002127; doi:10.1371/journal.pntd.0006562)
Supplement: S1 Text — (DOCX) [file pntd.0006562.s002.docx]

**Text S1.** The given formula follows from the definition of the cure rate.

True prevalence is reduced by the number of cured individuals between baseline and follow up in the case of no reinfections.

$$\pi_{FU,True}=(1-{CR}_{True})\pi_{BL,True}$$

The observed cure rate is equivalently defined

$$\pi_{FU,OBS}=s_{FU}\pi_{FU,True}=\left( 1-{CR}_{OBS} \right){s_{BL}\pi}_{BL,True}=\left( 1-{CR}_{OBS} \right)\pi_{BL,OBS}$$

Dividing the second equation by the first we get

$$s_{FU}=\frac{s_{FU}\pi_{FU,True}}{\pi_{FU,True}}=\frac{\left( 1-{CR}_{OBS} \right){s_{BL}\pi}_{BL,True}}{(1-{CR}_{True})\pi_{BL,True}}=\frac{\left( 1-{CR}_{OBS} \right)s_{BL}}{(1-{CR}_{True})}$$

Solving for the true cure rate leads to the equation given in the method section

$$\left( 1-{CR}_{True} \right)=\left( 1-{CR}_{OBS} \right)\frac{s_{BL}}{s_{FU}}$$
