# Supplementary material for: Diagnostic comparison between FECPAKG2 and the Kato-Katz method for analyzing soil-transmitted helminth eggs in stool
Source: PLoS Negl Trop Dis. 2018 Jun 4;12(6):e0006562. doi: 10.1371/journal.pntd.0006562 (PMC6002127; doi:10.1371/journal.pntd.0006562)
Supplement: S1 Table — (DOCX) [file pntd.0006562.s003.docx]

**S2 Table.** Estimated true prevalence, sensitivities, sensitivity-ratio and egg counts from the 231 participants with complete follow-up data according to the four different methods.

|  |  | *A. lumbricoides* | Hookworm | *T. trichiura* |
| --- | --- | --- | --- | --- |
| Estimated true | Prevalence | 5.5 (4.0-8.5) | 44.3 (39.4-50.5) | 52.0 (49.8-54.7) |
|  | Eggs per gram of stool | 5175 (2309-9567) | 179 (126-253) | 874 (638-1174) |
| Single Kato-Katz | No. of positive participants (%) | 5 (2.2) | 55 (23.8) | 97 (42.0) |
|  | Eggs per gram of stool | 1363 (-618-3344) | 190 (99-282) | 1211 (668-1754) |
|  | Sensitivity | 38.3 (24.7-57.6) | 58.1 (50.5-65.8) | 79.1 (72.7-87.1) |
|  | Sensitivity-ratio | 2.39 (1.66-3.74) | 1.46 (1.23-1.73) | 1.08 (1.00-1.18) |
| Duplicate Kato-Katz | No. of positive participants | 6 (2.6) | 67 (29.0) | 101 (43.7) |
|  | Eggs per gram of stool | 1140 (-463-2'743) | 148 (80-217) | 1159 (655-1'663) |
|  | Sensitivity | 51.8 (32.5-69.7) | 68.4 (59.6-76.5) | 85.9 (70.7-99.9) |
|  | Sensitivity-ratio | 1.69 (1.19-2.73) | 1.30 (1.12-1.54) | 1.14 (1.04-1.27) |
| Quadruplicate Kato-Katz | No. of positive participants | 7 (3.0) | 84 (36.4) | 112 (48.5) |
|  | Eggs per gram of stool | 971 (-342-2284) | 141 (50-232) | 1078 (589-1568) |
|  | Sensitivity | 60.5 (37.9-81.3) | 85.2 (74.3-95.3) | 97.1 (79.2-99.9) |
|  | Sensitivity-ratio | 1.57 (1.10-2.49) | 1.16 (1.01-1.36) | 1.04 (0.96-1.14) |
| FECPACK^G2^ | No. of positive participants | 4 (1.7) | 37 (16.0) | 49 (21.2) |
|  | Eggs per gram of stool | 366 (6-725) | 139 (61-217) | 164 (87-240) |
|  | Sensitivity | 52.9 (33.1-71.2) | 31.4 (27.4-35.1) | 34.2 (32.4-35.6) |
|  | Sensitivity-ratio | 1.38 (0.98-2.28) | 2.21 (1.88-2.63) | 2.06 (1.83-2.36) |
