# Supplementary material for: Diagnostic comparison between FECPAKG2 and the Kato-Katz method for analyzing soil-transmitted helminth eggs in stool
Source: PLoS Negl Trop Dis. 2018 Jun 4;12(6):e0006562. doi: 10.1371/journal.pntd.0006562 (PMC6002127; doi:10.1371/journal.pntd.0006562)
Supplement: S2 Table — (DOCX) [file pntd.0006562.s004.docx]

**S3 Table.** FECPAK^G2^ sensitivity according to the true infection intensity.

| Parasite | True eggs per gram of stool | Sensitivity (%) |
| --- | --- | --- |
| *A. lumbricoides* | 100 | 42.9 (37.3-46.9) |
|  | 200 | 52.7 (48.9-58.4) |
|  | 400 | 61.3 (57.0-66.1) |
|  | 800 | 68.5 (64.0-72.7) |
|  | 1000 | 70.6 (66.7-75.1) |
|  | 2000 | 76.2 (72.1-79.9) |
|  | 4000 | 80.8 (77.6-83.2) |
|  | 5000 - moderate infection intensity | 82.0 (78.8-84.5) |
| Hookworm | 100 | 56.3 (51.0-61.3) |
|  | 200 | 71.4 (66.6-75.2) |
|  | 400 | 82.8 (79.2-86.0) |
|  | 800 | 90.2 (87.8-92.9) |
|  | 1000 | 91.9 (90.1-94.7) |
|  | 2000 - moderate infection intensity | 95.6 (94.1-97.3) |
|  | 4000 - heavy infection intensity | 97.6 (95.9-98.6) |
|  | 5000 | 98.0 (97.1-99.1) |
| *T. trichiura* | 100 | 22.2 (19.9-23.5) |
|  | 200 | 37.1 (35.8-41.1) |
|  | 400 | 52.3 (49.5-55.9) |
|  | 800 | 66.3 (63.7-70.1) |
|  | 1000- moderate infection intensity | 70.3 (67.6-73.9) |
|  | 2000 | 80.5 (77.1-82.9) |
|  | 4000 | 87.3 (84.8-89.6) |
|  | 5000 | 90.8 (88.4-93.0) |
