# Supplementary material for: Diagnostic comparison between FECPAKG2 and the Kato-Katz method for analyzing soil-transmitted helminth eggs in stool
Source: PLoS Negl Trop Dis. 2018 Jun 4;12(6):e0006562. doi: 10.1371/journal.pntd.0006562 (PMC6002127; doi:10.1371/journal.pntd.0006562)
Supplement: S3 Table — (DOCX) [file pntd.0006562.s005.docx]

**S4 Table.** Estimated true and apparent cure rates (CR) according to the different diagnostic techniques and treatments arms.

| Diagnostic method | Tribendimidine | | | | Tribendimidine-ivermectin | | | | Tribendimidine-oxantel pamoate | | | | Albendazole-oxantel pamoate | | | |
| --- | --- | --- | --- | --- | --- | --- | --- | --- | --- | --- | --- | --- | --- | --- | --- | --- |
|  | No. positive | Total No. | CR (%) | 95%-CI | No. positive | Total No. | CR (%) | 95%-CI | No. positive | Total No. | CR (%) | 95%-CI | No. positive | Total No. | CR (%) | 95%-CI |
| ***Ascaris lumbricoides*** | | | | | | | | | | | | | | | | |
| True CRs |  |  | 92.2 | 85.2-94.9 |  |  | 93.5 | 82.8-97.3 |  |  | 93.1 | 87.9-95.6 |  |  | 92.3 | 85.2-95.5 |
| Single Kato-Katz | 0 | 33 | 100.0 | - | 0 | 36 | 100.0 | - | 1 | 38 | 97.4 | 86.2-99.9 | 2 | 46 | 95.7 | 85.2-99.5 |
| Duplicate Kato-Katz | 0 | 33 | 100.0 | - | 1 | 36 | 97.2 | 85.5-99.9 | 1 | 39 | 97.4 | 86.5-99.9 | 2 | 46 | 95.7 | 85.2-99.5 |
| Quadruplicate Kato-Katz | 1 | 36 | 97.2 | 85.5-99.9 | 1 | 38 | 97.4 | 86.2-99.9 | 1 | 45 | 97.8 | 88.2-99.9 | 2 | 47 | 95.7 | 85.5-99.5 |
| FECPAK^G2^ | 1 | 25 | 96.0 | 79.6-99.9 | 0 | 34 | 100.0 |  | 1 | 34 | 97.1 | 84.7-99.9 | 2 | 37 | 94.6 | 81.8-99.3 |
| **Hookworm** | | | | | | | | | | | | | | | | |
| True CRs |  |  | 47.3 | 26.8-56.7 |  |  | 77.0 | 62.7-83.6 |  |  | 46.3 | 35.2-52.6 |  |  | 49.2 | 36.7-56.2 |
| Single Kato-Katz | 11 | 44 | 75.0 | 59.7-86.8 | 6 | 46 | 87.0 | 73.7-95.1 | 17 | 55 | 69.1 | 55.2-80.9 | 14 | 47 | 70.2 | 55.1-82.7 |
| Duplicate Kato-Katz | 16 | 46 | 65.8 | 49.8-78.6 | 6 | 48 | 87.5 | 74.8-95.3 | 20 | 58 | 65.5 | 51.9-77.5 | 18 | 50 | 64.0 | 49.2-77.1 |
| Quadruplicate Kato-Katz | 19 | 50 | 62.0 | 47.2-75.3 | 9 | 56 | 83.9 | 71.7-92.4 | 28 | 61 | 54.1 | 40.8-66.9 | 25 | 58 | 56.9 | 43.2-69.8 |
| FECPAK^G2^ | 12 | 34 | 64.7 | 46.5-80.3 | 3 | 38 | 92.1 | 78.6-98.3 | 8 | 46 | 82.6 | 68.6-92.2 | 7 | 40 | 82.5 | 67.2-92.7 |
| ***Trichuris trichiura*** | | | | | | | | | | | | | | | | |
| True CRs |  |  | 5.5 | 1.6-8.5 |  |  | 34.1 | 25.7-37.7 |  |  | 66.8 | 58.1-71.1 |  |  | 74.0 | 67.4-77.3 |
| Single Kato-Katz | 6 | 48 | 12.5 | 4.7-25.2 | 26 | 56 | 53.6 | 39.7-67.0 | 16 | 60 | 73.3 | 60.3-83.9 | 11 | 54 | 79.6 | 66.5-89.4 |
| Duplicate Kato-Katz | 43 | 48 | 10.4 | 3.5-22.7 | 28 | 57 | 50.9 | 37.3-64.4 | 16 | 60 | 73.3 | 60.3-83.9 | 11 | 54 | 79.6 | 66.5-89.4 |
| Quadruplicate Kato-Katz | 44 | 49 | 10.2 | 3.4-22.2 | 35 | 57 | 38.6 | 26.0-52.4 | 19 | 61 | 68.9 | 55.7-80.1 | 12 | 57 | 78.9 | 66.1-88.6 |
| FECPAK^G2^ | 23 | 34 | 32.4 | 17.4-50.5 | 9 | 38 | 76.3 | 59.8-88.6 | 3 | 41 | 92.7 | 80.1-98.5 | 6 | 42 | 85.7 | 71.5-94.6 |
