# Supplementary material for: Diagnostic comparison between FECPAKG2 and the Kato-Katz method for analyzing soil-transmitted helminth eggs in stool
Source: PLoS Negl Trop Dis. 2018 Jun 4;12(6):e0006562. doi: 10.1371/journal.pntd.0006562 (PMC6002127; doi:10.1371/journal.pntd.0006562)
Supplement: S4 Table — (DOCX) [file pntd.0006562.s006.docx]

**S5 Table.** Estimated true arithmetic egg reduction rates (ERR) and apparent ERRs by the single (KK1), duplicate (2KK), quadruplicate Kato-Katz (4KK) and FECPAK^G2^ (FP).

| Method | Tribendimidine | | | | | | Tribendimidine-ivermectin | | | | | Tribendimidine-oxantel pamoate | | | | | Albendazole-oxantel pamoate | | | | |
| --- | --- | --- | --- | --- | --- | --- | --- | --- | --- | --- | --- | --- | --- | --- | --- | --- | --- | --- | --- | --- | --- |
|  | N | EPG pre | EPG post | ERR (%) | 95%-CI | N | | EPG pre | EPG post | ERR (%) | 95%-CI | N | EPG pre | EPG post | ERR (%) | 95%-CI | N | EPG pre | EPG post | ERR (%) | 95%-CI |
| ***Ascaris lumbricoides*** | | | | | | | | | | | | | | | | | | | | | |
| True |  | 11437 | 323 | 97.3 | 91.3-99.9 |  | | 9’141 | 139 | 98.4 | 93.2-99.9 |  | 9673 | 258 | 97.3 | 92.9-99.5 |  | 15235 | 399 | 97.3 | 92.0-99.4 |
| KK1 | 33 | 13237 | 0 | 100.0 | - | 36 | | 19296 | 0 | 100.0 | - | 38 | 11949 | 19 | 99.8 | 99.3-100.0 | 46 | 16573 | 131 | 99.2 | 97.7-100.0 |
| KK2 | 33 | 13248 | 0 | 100.0 | - | 36 | | 19066 | 0.7 | >99.9 | >99.9-100.0 | 39 | 12068 | 18 | 99.9 | 99.3-100.0 | 46 | 16616 | 133 | 99.2 | 97.5-100.0 |
| KK4 | 36 | 14048 | 0.2 | >99.9 | >99.9-100.0 | 38 | | 16294 | 0.3 | >99.9 | >99.9-100.0 | 45 | 11547 | 20 | 99.8 | 99.3-100.0 | 47 | 17434 | 124 | 99.3 | 97.9-100.0 |
| FP | 25 | 3565 | 7 | 99.8 | 99.3-100.0 | 34 | | 2962 | 0 | 100.0 | - | 34 | 3234 | 16 | 99.5 | 97.9-100.0 | 37 | 4168 | 20 | 99.5 | 98.3-100.0 |
| **Hookworm** | | | | | | | | | | | | | | | | | | | | | |
| True |  | 545 | 155 | 72.6 | 47.3-90.1 |  | | 494 | 29 | 95.8 | 85.0-99.0 |  | 370 | 81 | 81.9 | 66.3-90.9 |  | 577 | 62 | 92.4 | 84.9-96.7 |
| KK1 | 44 | 574 | 118 | 79.4 | 63.5-93.9 | 46 | | 584 | 7 | 98.8 | 97.7-99.6 | 55 | 515 | 43 | 91.7 | 81.7-96.7 | 47 | 625 | 39 | 93.8 | 89.6-97.5 |
| KK2 | 46 | 558 | 102 | 81.7 | 66.1-94.2 | 48 | | 537 | 7 | 98.7 | 97.6-99.7 | 58 | 447 | 47 | 89.5 | 79.6-95.0 | 50 | 570 | 33 | 94.2 | 90.5-97.5 |
| KK4 | 50 | 516 | 130 | 74.8 | 45.9-93.3 | 56 | | 452 | 8 | 98.2 | 97.2-99.4 | 61 | 403 | 50 | 87.6 | 78.8-92.8 | 58 | 553 | 30 | 94.6 | 91.3-97.1 |
| FP | 34 | 357 | 85 | 76.2 | 49.3-94.2 | 38 | | 215 | 6 | 97.2 | 94.2-100.0 | 46 | 316 | 18 | 94.3 | 88.0-98.4 | 40 | 209 | 20 | 90.4 | 77.9-97.6 |
| ***Trichuris trichiura*** | | | | | | | | | | | | | | | | | | | | | |
| True |  | 2944 | 2242 | 22.9 | 5.3-50.3 |  | | 1627 | 162 | 91.0 | 83.7-95.9 |  | 1801 | 110 | 94.3 | 88.1-97.5 |  | 2796 | 114 | 96.1 | 90.3-98.6 |
| KK1 | 48 | 2729 | 2151 | 21.2 | -9.4-41.4 | 56 | | 1800 | 84 | 95.3 | 91.8-97.1 | 60 | 1891 | 74 | 96.1 | 91.6-98.6 | 54 | 2245 | 92 | 95.9 | 90.4-99.3 |
| KK2 | 48 | 2732 | 2128 | 22.1 | -6.4-43.1 | 57 | | 1625 | 88 | 94.6 | 90.4-96.8 | 60 | 1821 | 71 | 96.1 | 91.7-98.6 | 54 | 2188 | 103 | 95.3 | 88.6-99.3 |
| KK4 | 49 | 2596 | 2140 | 17.6 | -17.1-38.8 | 57 | | 1719 | 114 | 93.4 | 87.6-96.3 | 61 | 1786 | 86 | 95.2 | 89.6-98.4 | 57 | 2292 | 74 | 96.8 | 93.0-99.4 |
| FP | 34 | 255 | 180 | 29.4 | -38.3-66.7 | 38 | | 190 | 15 | 92.1 | 84.4-96.3 | 41 | 163 | 7 | 95.7 | 88.9-100.0 | 42 | 145 | 10 | 93.1 | 84.9-98.4 |
